# Adaptation of conventional water treatment technologies for organic component removal from liquid radioactive waste: sorption and coagulation mechanisms

**DOI:** 10.1038/s41598-026-36799-2

**Published:** 2026-01-20

**Authors:** Dmytro Charnyi, Yuriy Zabulonov, Vitalina Lukianova, Yevheniia Anpilova, Nataliia Chernova, Yevhen Matselyuk, Serhiy Marisyk

**Affiliations:** 1 State Institution “The Institute of Environmental Geochemistry of National Academy of Sciences of Ukraine”, Academician Palladin Avenue, 34а, Kyiv-142, 03142 Ukraine; 2 National University of Food Technologies, Volodymyrska str., 68, Kyiv, 01601 Ukraine; 3 Institute of Water Problems and Land Reclamation of the National Academy of Agrarian Sciences of Ukraine, Vasylkivska, Str., 37, Kyiv, 03022 Ukraine; 4 Institute of Telecommunications and Global Information Space of the National Academy of Sciences of Ukraine, Chokolivskiy bulv., 13, Kyiv-186, 03186 Ukraine; 5 Helmholtz Centre for Environmental Research GmbH, Permoserstraße 15, 04318 Leipzig, Germany

**Keywords:** Liquid radioactive waste, Radionuclides, Organic matter, Activated carbon, Water treatment, Sorbents, Oxidation, Coagulation, Filtration, Chemistry, Environmental sciences, Materials science

## Abstract

Significant concentrations of artificial radionuclides have been detected in drinking water sources not only in areas contaminated by major radiation accidents, but also in the vicinity of operating nuclear power plants. Therefore, the use of effective, broad-spectrum sorbents produced from readily available Ukrainian raw materials in water treatment and purification technologies should be regarded as a strategically important social measure. However, technologies that rely on oxidation to decompose organic components require the recycling of this process. Other promising methods, such as plasma treatment, photocatalysis, and electrocatalysis, currently lack industrial-scale equipment. Meanwhile, there are established water treatment technologies that, despite their proven effectiveness, have not yet been adapted for the treatment of liquid radioactive waste (LRW). This paper investigates the potential application of conventional water treatment technologies for removing the organic component from LRW at nuclear power plants. By employing sorption (using activated carbon and powdered bentonite), coagulation (with ferric chloride), and filtration. We achieved a 75% removal efficiency of the organic component, as measured by COD(Cr) (dichromate oxidizability). The use of a 20% w/v FeCl₃ solution as a coagulant and activated carbon as a sorbent significantly enhanced the purification process. Reducing the concentration of bentonite had little effect on the degree of LRW purification, whereas increasing its concentration noticeably diminished purification efficiency. Nevertheless, the addition of bentonite as a turbidity agent substantially accelerated coagulation and sedimentation. Conversion models were developed to recalculate COD(Cr) (dichromate oxidizability) indicators into corresponding COD(Mn) (permanganate oxidizability) or total organic carbon (TOC), with prediction accuracy validated by experimental data. It should be noted that using such technologies will significantly reduce water and energy consumption, as well as saving time, all of which is extremely important in Ukraine’s current wartime economic situation.

## Introduction

The management of LRW containing organic substances is a critical challenge in the nuclear industry, research laboratories, and medical facilities. Organic compounds in LRW - such as solvents, oils, scintillation fluids, and decontamination agents - complicate treatment due to their chemical stability, radiolytic degradation, and potential for secondary waste generation. Effective removal and destruction of these organics are vital for environmental safety, regulatory compliance, and the long-term stability of conditioned waste forms.

Organic substances enter LRW streams from various sources^1–3^:

- Nuclear power plants (NPP): lubricants, oils, and solvents used in maintenance and decontamination;
- Research and medical facilities: scintillation fluids, laboratory solvents, and organic reagents;
- Decontamination processes: detergents, surfactants, and chelating agents.

These organics can be present as dissolved or emulsified compounds, suspended solids or sludges, and complexing agents that bind radionuclides, reducing the efficiency of standard removal techniques^1,4,5^. Their presence increases the complexity of waste management due to chemical and radiolytic stability, flammability and toxicity, interference with solidification and immobilization processes.

The most common method of treating LRW is evaporation. However, layers of vulcanized polymer deposits form on the inner surfaces of the evaporators during the evaporation process. This requires mechanical cleaning, which leads to additional personnel exposure and may cause the technological process to stop.

Evaporation results in the formation of a liquid radioactive concentrate, which is a colloidal system consisting of an aqueous phase and a solid phase (sediment) in the form of a pulp. The pulp contains finely dispersed mineral particles with an adsorption layer of undifferentiated organic substances. Direct cementation is impossible due to the low mobility of the sediments and the presence of organic impurities, which must be removed first. However, current methods for removing organic substances are not very effective.

Physicochemical methods remain the cornerstone of LRW treatment for organic contaminants. Advances in oxidation, membrane, and hybrid technologies are improving efficiency and reducing environmental risks. Ongoing research and process integration are essential for addressing the diverse and evolving challenges posed by organic substances in radioactive waste.

Separation and concentration of organic components from LRW are achieved by physical and chemical methods^1,2,5^. Distillation effectively separates volatile organics but is unsuitable for thermally unstable or high-boiling compounds due to secondary residue formation^1,2^. Membrane processes (ultra-, microfiltration, reverse osmosis, electrodialysis) provide selective removal of colloids, dissolved organics, and radionuclides but are limited by fouling^3–8^. Adsorption and ion exchange using activated carbon, zeolites, resins, and biosorbents, as well as hybrid membrane–sorption systems, improve removal efficiency but face adsorbent saturation and secondary waste issues^4,5,9^. Destruction methods chemically or physicochemically degrade organics into less hazardous forms. Wet air and supercritical water oxidation achieve deep decomposition of complex compounds^1,2^; advanced oxidation processes (ozonation, Fenton, photocatalysis, plasma) generate radicals for rapid breakdown^5^; and electrochemical oxidation is suitable for small, high-activity waste streams^2,10^.

Immobilization involves combined or hybrid physical, chemical, and biological treatments to minimize secondary waste^4–9,11,12^. Industrial examples include wet air oxidation in Winfrith (UK) and the ELIPSE plasma system in France^1,2^. Main challenges remain secondary waste, material corrosion, and process cost^1,2,5^. Promising advances include selective sorbents (MOFs, biosorbents), modular plants, and automated control systems^1,4–9,13^.

Technologies for the removal of LRW must be sufficiently reliable, well-tested, and at the same time relatively inexpensive.

Currently, in the modern nuclear power industry, ion-exchange and membrane technologies are the most widely used, whereas classical technologies based on accessible and well-established methods of municipal water treatment are hardly considered.

In any case, the authors have not found any recent sources reporting the testing of such approaches, which is one of the reasons that prompted them to initiate this work precisely in the direction of the classical adsorption–coagulation–filtration technology, followed by cementation of the obtained sludge as a component of alkali-activated concrete.

In Ukraine, nuclear power accounts for 51.2% of total electricity generation (76.2 TWh in 2019)^14^, and over 20,000 m^3^ of low-level radioactive waste is stored at the Chernobyl site. The limited availability of freshwater (approximately 1200 m^3^ per capita in dry years) and the use of Dnipro River basin waters for cooling 12 reactors and supplying 65% of drinking water exacerbate ecological risks^15,16^.

In the context of Ukraine’s wartime economy, constrained energy and water supplies, and the urgent need for cost-effective decontamination technologies, the development of simple, low cost and environmentally sustainable methods for LRW treatment becomes especially important. The authors therefore aimed to demonstrate practical and affordable technologies for processing liquid radioactive waste that can be implemented under resource-limited conditions. Such approaches are not only relevant for Ukraine but also for other countries that already operate nuclear technologies or plan to develop and deploy small modular reactors (SMRs) in the near future.

The presence of organic compounds in wastewater reduces the efficiency of radionuclide removal by four to five times^17^; highly alkaline conditions (pH 11–13) make ozonation difficult, often necessitating multiple cycles^18–20^. An alternative to ozonation is the oxidation of synthetic surfactants using electric discharge plasma treatment of wastewater. This method is promising, as evidenced by the higher oxidation potential of hydroxyl radicals (2.7 V), generated during plasma treatment, compared to ozone (2.07 V)^21,22^. The use of oxidation technologies based on the Fenton reaction is not feasible due to the high pH levels^23–27^. In our view, combined membrane-sorption methods for removing organic compounds are also promising^28–31^, as are photocatalytic and electrocatalytic approaches^32,33^. However, almost all of these methods currently have no commercially available serial equipment.

Although classical water treatment methods for removing organic components, such as sorption and coagulation^34–36^, have been well developed and typically achieve removal rates of no less than 70–80%, they have not yet been widely applied in wastewater treatment technologies for nuclear facilities.

The obtained results indicate that the combined application of adsorption and coagulation processes can achieve a substantial reduction in the organic component of model liquid radioactive waste. The correlation analysis confirmed that the efficiency of purification strongly depends on the type and dosage of sorbents and coagulants, while an excessive increase in bentonite concentration can negatively affect the removal efficiency due to the stabilisation of colloidal particles. These findings suggest that the optimisation of reagent composition and process parameters plays a decisive role in improving the overall decontamination performance.

Thus, the authors concluded that most contemporary approaches rely on technologies employing various oxidising agents produced through different chemical or electrochemical methods. However, these processes share a common limitation: they are energy-intensive and, more importantly, remain at the experimental or pre-industrial stage of development. Their large-scale implementation would require substantial financial investment and a high level of technological innovation.

In contrast, this opens a promising direction for the application of conventional water treatment technologies, which have long been established and validated in other engineering fields. Appropriate optimisation, such as selecting specific reagents, adjusting operational parameters and integrating sorption or membrane stages, can make classical approaches cost-effective, reliable and environmentally sustainable alternatives for treating radioactive liquid waste.

## Materials and methodology

The aim of this research was to investigate the removal of the organic fraction from LRW using a combined approach that integrates adsorption, coagulation, and filtration. The study employed reagents that are commonly used in conventional water treatment processes: activated carbon as the sorbent; powdered bentonite as the turbidity-inducing agent; ferric chloride as the coagulant; and filter paper as the filtration medium.

The experiments were conducted using a model solution designed to simulate the organic fraction of LRW. To prepare the concentrated model solution, tap water was first boiled and allowed to stand to facilitate carbonate precipitation. The following components were then added to obtain 5 L of solution:

1. 3.5 g of hydrazine;
2. 3.25 g of oxalic acid;
3. 0.5 g of citric acid;
4. 0.5 g of potassium permanganate (KMnO₄);
5. 9.5 g of the powder phosphate surfactant;
6. 0.5 g of Trilon B;
7. 7.5 g of liquid soap.

The solution was thoroughly mixed and left to stand for 24 h. In experiments we used the diluted concentrated model solution by 10 times.

Activated carbon was added to the model solution as a powdered carbonaceous sorbent (Table 1).

**Table 1:** Particle size properties of coconut activated carbon.

Size, mm	Share, %
0.63	4.0
0.5	11.6
0.4	27.4
0.315	39.6
0.2	15.6
< 0.2	1.8
Moisture, %.	5.88
Ash, %.	3.87
pH of the water extract	9.7
Iodine number, mg/g (ASTM)	1269

**Fig. 1:**
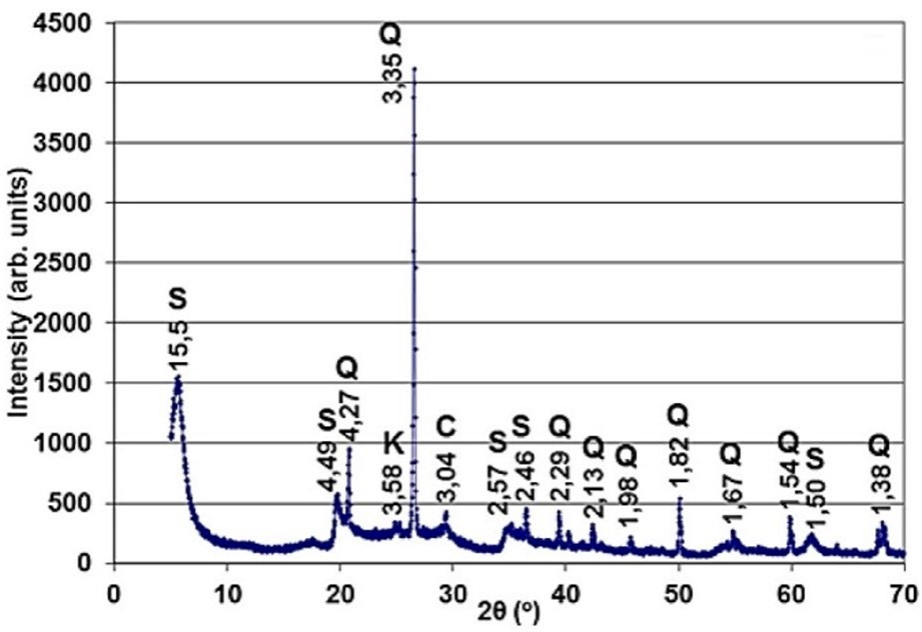
Diffractogram of bentonite clays from the Dashukivka deposit^39^ was reproduced with permission and published under the Creative Commons Attribution 4.0 International License (CC BY 4.0).

Industrial-scale natural bentonite clays were sourced from the Cherkasy deposit of bentonite and palygorskite clays, which is currently the largest in Ukraine—accounting for approximately 80% of the country’s bentonite reserves—and one of the largest in Europe. The primary extraction occurs at the Dashukivka deposit of Cherkasy region, covering an area of about 2.7 km^2^, which was thoroughly explored between 1958 and 1960. Physicochemical properties and mineralogical composition of bentonite presented in Tables 2, 3, and 4^37,38^ justify its high ion exchange capacity determined both theoretically and experimentally (70 and 74 meq/100 g correspondingly) and interlayer charge. Diffractogram of bentonite clays from the Dashukivka deposit^39^ was presented in Fig. 1. This image of the diffractogram was obtained and stored at the State Institution “Institute of Environmental Geochemistry of the National Academy of Sciences of Ukraine” with permission and published under the Creative Commons Attribution 4.0 International License (CC BY 4.0).

The interaction energy between exchangeable cations and clay-mineral surfaces is contingent on the cations’ ionic size, oxidation state, and the charge distribution in the Smectite lattice. Charges from ionic substitution in the tetrahedral layer are located on the outer surfaces, whereas those from the octahedral layer are situated deeper within the particle. Consequently, the cations that balance the octahedral layer charge exhibit a weaker binding affinity compared to those found in the tetrahedral layer^40^.

Heterovalent cation exchange affects unit-cell parameters and is the basis of cation exchange capacity (CEC), influencing rheology and adsorption. The strong negative charge on the tetrahedral layer has been demonstrated to be effective in preventing aggregation and promoting dispersion of large particles. The CEC is primarily attributable to ion substitution (~ 80%) and the remainder (~ 20%) to bond cleavage at edges, where hydroxyl groups – initially bound to Al^3+^ or Mg^2+^ in the octahedral layer – mediate exchange^41,42^.

The crystallochemical formula of the dioctahedral smectites from the Dashukivka bentonite deposit is as follows^37^:

$$\:\left({Al}_{1.21}{{Fe}^{3+}}_{\:\mathrm{0,49}}{Mg}_{0.30}\right)\left[{Al}_{0.18}{Si}_{3.82}\right]{O}_{10}(OH{)}_{2}+({Ca}_{0.17}{Na}_{0.03})$$

**Table 2:** The chemical composition of the bentonite from Dashukivka deposit determined by silicate chemical analysis^37^.

Chemical compound	wt %
$\:{SiO}_{2}$	50.60
$\:{Al}_{2}{O}_{3}$	15.58
$\:{Fe}_{2}{O}_{3}$	8.72
$\:{TiO}_{2}$	0.50
$\:MgO$	2.64
$\:CaO$	2.07
$\:{Na}_{2}O$	0.20
$\:{H}_{2}O\left(+\right)$	6.26
$\:{H}_{2}O\left(-\right)$	13.46
LOI*	19.72
Sum	100.30

*Loss on ignition (LOI - %) represents the determination of the mass change of a sample after heating to a defined temperature, resulting from the evaporation or combustion of volatile components Eq. (1). In this study, the gravimetric method was applied within the temperature range of 103–105 °C to determine the content of moisture and volatile matter in the sample.

1 $$\:\mathrm{LOI\:(\%)}=\frac{{m}_{i}-{m}_{f}}{{m}_{i}}\times\:100\mathrm{\%}$$

where $\:{m}_{i}$ - initial mass (g) is the weight of the sample before heating, and the $\:{m}_{f}$ - final mass (g) is the weight of the sample after heating.

**Table 3:** Mineral composition of natural bentonite samples from the Dashukivka deposit (X-ray phase analysis data), Wt %^37^.

Mineral	wt %
Smectite	65–70
Quartz	20–25
Calcite	3–5
Kaolinite	3–5
Mica	5
Feldspars	3
Cristobalite	Not observed
Anataz	3

**Table 4:** Total exchange capacities of cations, charge values of octahedral and tetrahedral layers (Z), total interlayer charge (ΣZ) of smectite minerals of the Dashukivka deposit, meq/100 g of the mineral^37^.

Layer	Z, mV
Tetrahedron	− 0.179
Octahedron	− 0.185
Tolal interlayer charge (ΣZ)	− 0.364
Total CEC	70

Specific surface of Dashukivka’s bentonite measured by water and hexane adsorption is 375 and 69 $\:m/g$ correspondently^37^.

**Fig. 2:**
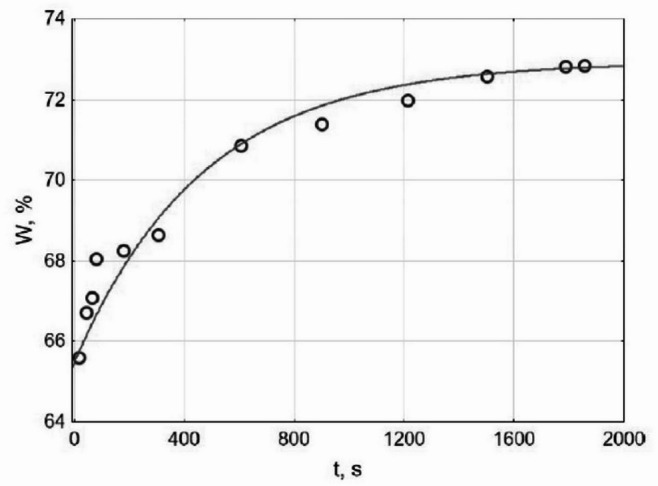
Decane sorbtion by smectites, W, wt % (Dashukivka deposit): model applicated to experimental study (plotted by points)^37^.

An important parameter is the porosity of bentonite. The relative value of decane sorption (*W*, wt %) is closely associated with the porosity of natural minerals, which, according to experimental studies of Dashukivka’s bentonite, exceeds 70% (see Fig. 2). Notably, the filling of macropores (approximately 65%) occurs almost instantaneously within the first seconds of contact between the solid and liquid phases. Subsequent sorption of decane into the micropores and interstructural packets of the mineral (about 8%) proceeds much more slowly and can be described in terms of chemical kinetics. Given the experimentally determined direction of the process, it is appropriate to apply kinetic laws for an irreversible process, specifically the first-order kinetic Eq. (2):

2 $$\:W={W}_{0}+{W}_{1}\left(1-{e}^{-kt}\right),$$

where $\:{W}_{0}$ (wt %) and $\:{\:W}_{1}$ (wt %) represent the sorption of decane in macropores (65.5%) and in micropores and interstructural packets of the mineral (7.44%), repectively; *k* is the rate constant for sorption in micropores and interstructural packets of the mineral (0.00213 $\:{s}^{-1}$); and *t* – time (s) elapsed since the beginning of the experiment. The correlation coefficient for fitting the kinetic model to the experimental data presented and shown^37^ in Fig. 2 is 0.98.

General statistical approaches to regression equations were applied for model development, and the following open-source software was used:

- PSPP, which is licensed under the GNU General Public License (GPLv3 or later);
- Scilab, which is licensed under the CeCILL License and is compatible with GPL.

The experimental data are best described by a two-site model assuming instantaneous filling of macropores and kinetic filling of micropores. The kinetic part follows a pseudo-first-order (Lagergren-type) rate equation, showing a rapid initial uptake and gradual saturation typical of systems combining fast and slow processes. This two-fraction approach provides the most adequate description of the observed behavior.

## Experimental

The activated coconut carbon was in the form of a fine powder. To prepare a suspension, 5 g of activated carbon was added to 30 ml of distilled water. The mixture was stirred and boiled for 3 min to allow water to penetrate the pores of the carbon. This process reduced the positive buoyancy of the carbon particles.

**Fig. 3:**
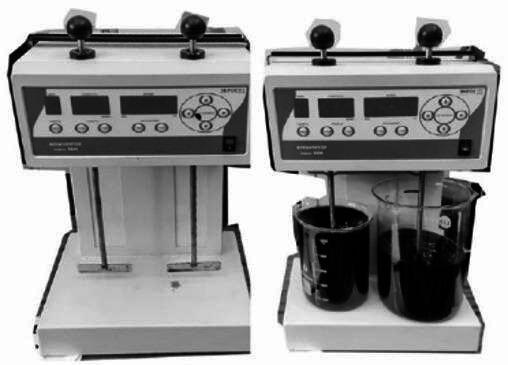
Experimental research setup: laboratory flocculator (PE-8800, Ekros).

Then, when the activated carbon slurry had a temperature of 25 °C, it was mixed using a PE-8800 laboratory flocculator (Ekros) (Fig. 3). The adjustable rotation speed of the flocculator PE-8800 (Ekros) ranges from 20 to 350 rpm, allowing flexible control of the mixing intensity of water samples according to the requirements of the experiment or technological process. In our case, the rotation speed was set to 50 rpm.

The mixture was stirred for 20 min to ensure adequate contact between the activated carbon and the solution. After the activated carbon slurry had been mixed, powdered bentonite in doses ranging from 1 to 10 g/L was introduced, followed by a further 20 min of stirring.

**Fig. 4:**
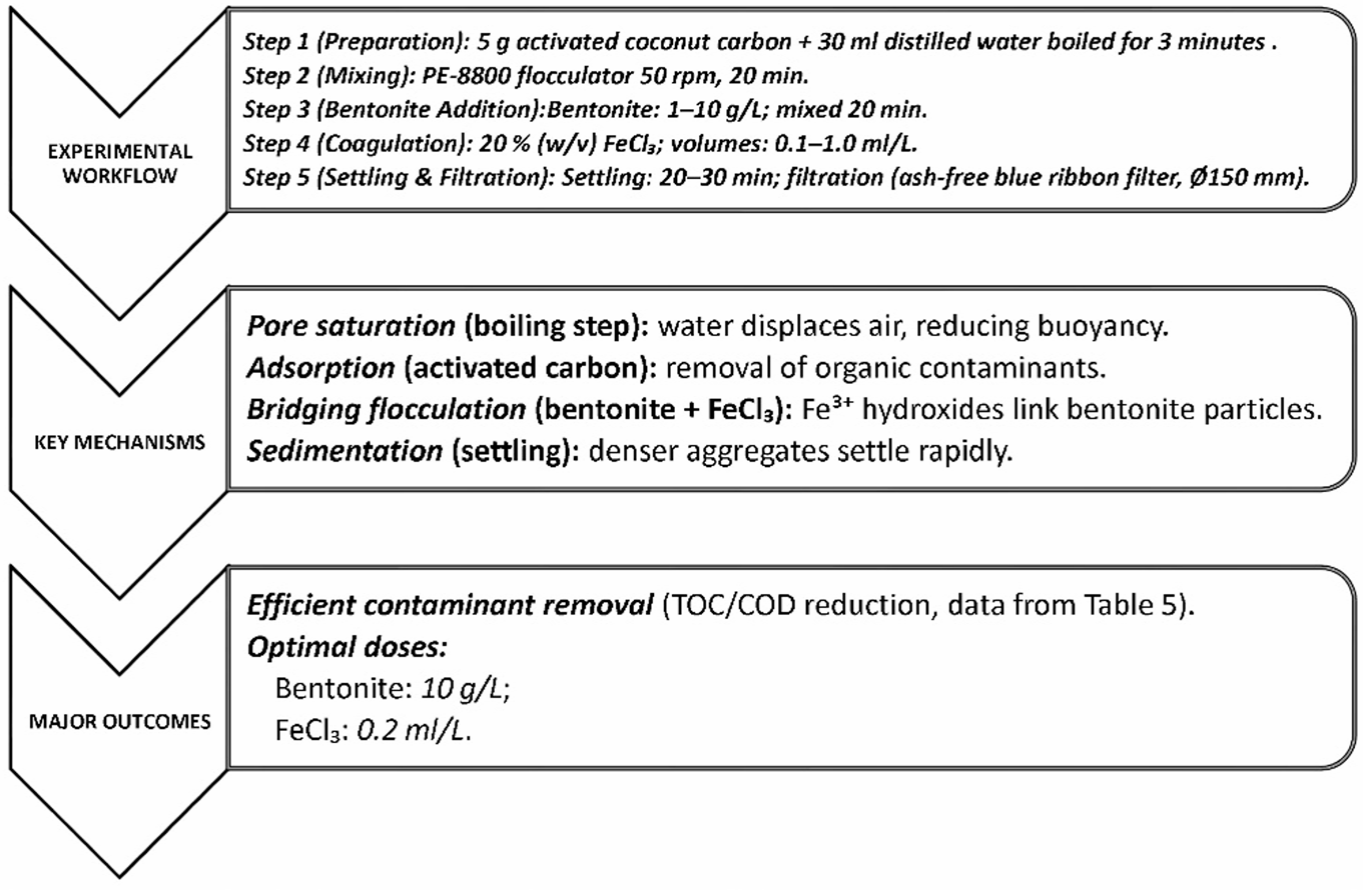
Stepwise treatment process.

The coagulant (20% (w/v) FeCl_3_) was then added to the mixture, which was gently stirred for 40 s. The volume of ferric chloride solution used for the coagulation process was determined through visual assessment. The volumes of FeCl_3_ solution added per 1 ml of bentonite suspension containing 10 g/L of bentonite were 1.0, 0.5, 0.3, 0.2 and 0.1 ml respectively. The most effective sedimentation of bentonite was visually achieved with the addition of a 0.2 ml/L ferric chloride solution (see Table 5).

The suspension was left to settle for 20–30 min and subsequently filtered (ash-free filter paper, Ø150 mm, blue ribbon type).

The stepwise treatment procedure and underlying mechanisms are illustrated in Fig. 4.

**Table 5:** Analysis of surfactant-coagulated samples and experimental results on the reduction of the organic fraction in LRW simulants.

Sample no.	Treatment agents			Organic pollution		
	Activated carbon (calculated on a dry basis), $\:\mathrm{g}/\mathrm{L}$	20% w/v FeCl₃, $\:\mathrm{m}\mathrm{l}$	Bentonite, $\:\mathrm{g}/\mathrm{L}$	TOC, $\:\:\mathrm{m}\mathrm{g}\mathrm{C}/\mathrm{L}$	COD(Mn), $\:\mathrm{m}\mathrm{g}{O}_{2}/\mathrm{L}$	COD(Cr), $\:\mathrm{m}\mathrm{g}{O}_{2}/\mathrm{L}$
0	0	0	0	27.2	29.44	83.7
1	5	0.2	10	14.9	14.72	36
2	5	0.2	7	14.8	14.72	35
3	5	0.2	4	13.4	14.40	27.8
4	5	0.2	3	12.4	14.40	24.5
5	5	0.2	2	12.6	13.92	24.7
6	5	0.2	1	9.53	11.20	20.7

The following characteristics were controlled in the experimental solutions: Total Organic Carbon (TOC), Dichromate COD (COD(Cr)), Permanganate COD (COD(Mn))^37–45^.

TOC and COD(Mn) were determined in the main laboratory of the Production Monitoring and Technological Control Department of PJSC “Kyivvodokanal” (DSTU (State Standard of Ukraine) EN ISO/IEC 17025:2019 (EN ISO/IEC 17025:2017, IDT; ISO/IEC 17025:2017, IDT) “General requirements for the competence of testing and calibration laboratories”)^46^.

TOC was determined using a TOC-L CPN (Shimadzu) analyzer, based on the thermo-catalytic oxidation method at 680 °C. This technique enables complete oxidation of both easily degradable and refractory organic compounds without catalyst fouling. The TOC concentration was obtained by calculating the difference between total carbon (TC) and inorganic carbon (IC). The method covers a measurement range from 4 µg/L to 30 000 mg/L and ensures accurate and reproducible results for various water samples, from ultrapure to highly contaminated wastewater.

COD(Cr) was determined in the Laboratory of Environmental Quality Assessment of the State Institution “The Institute of Environmental Geochemistry of National Academy of Sciences of Ukraine”^47^ (Certificate of measurement capabilities recognition DSTU (State Standard of Ukraine) EN ISO 10012:2022 Quality management – Requirements for measurement management systems (EN ISO 10012:2003, IDT; ISO 10012:2003, IDT)^48^ according to the methods specified in List of measuring capabilities of the laboratory for assessing environmental quality parameters^49^.

The analysis was performed using the Hach Lange DR-2800 spectrophotometer in accordance with the certified dichromate method employing PERMACHEM reagent packs (Hach). This standardized photometric method ensures accurate, reliable, and reproducible determination of COD in water samples. The measurement accuracy of the spectrophotometer is ± 1% of the reading or ± 0.005 Abs, whichever is greater.

**Fig. 5:**
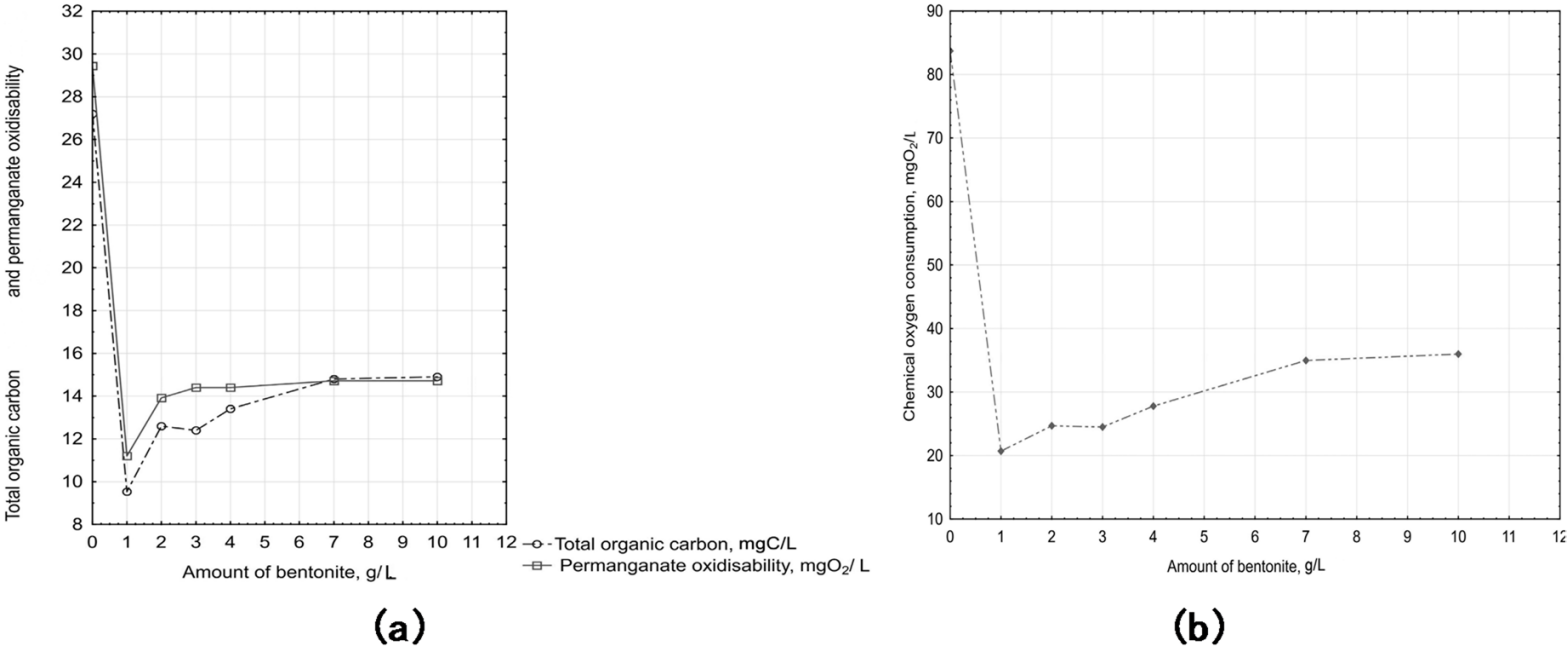
Dependence of organic component concentration: (**a**) TOC and COD(Mn); (**b**) COD(Cr) on the bentonite dosage.

The graphs (Fig. 5) show the dependence of changes in the concentration of the organic component.

**Fig. 6:**
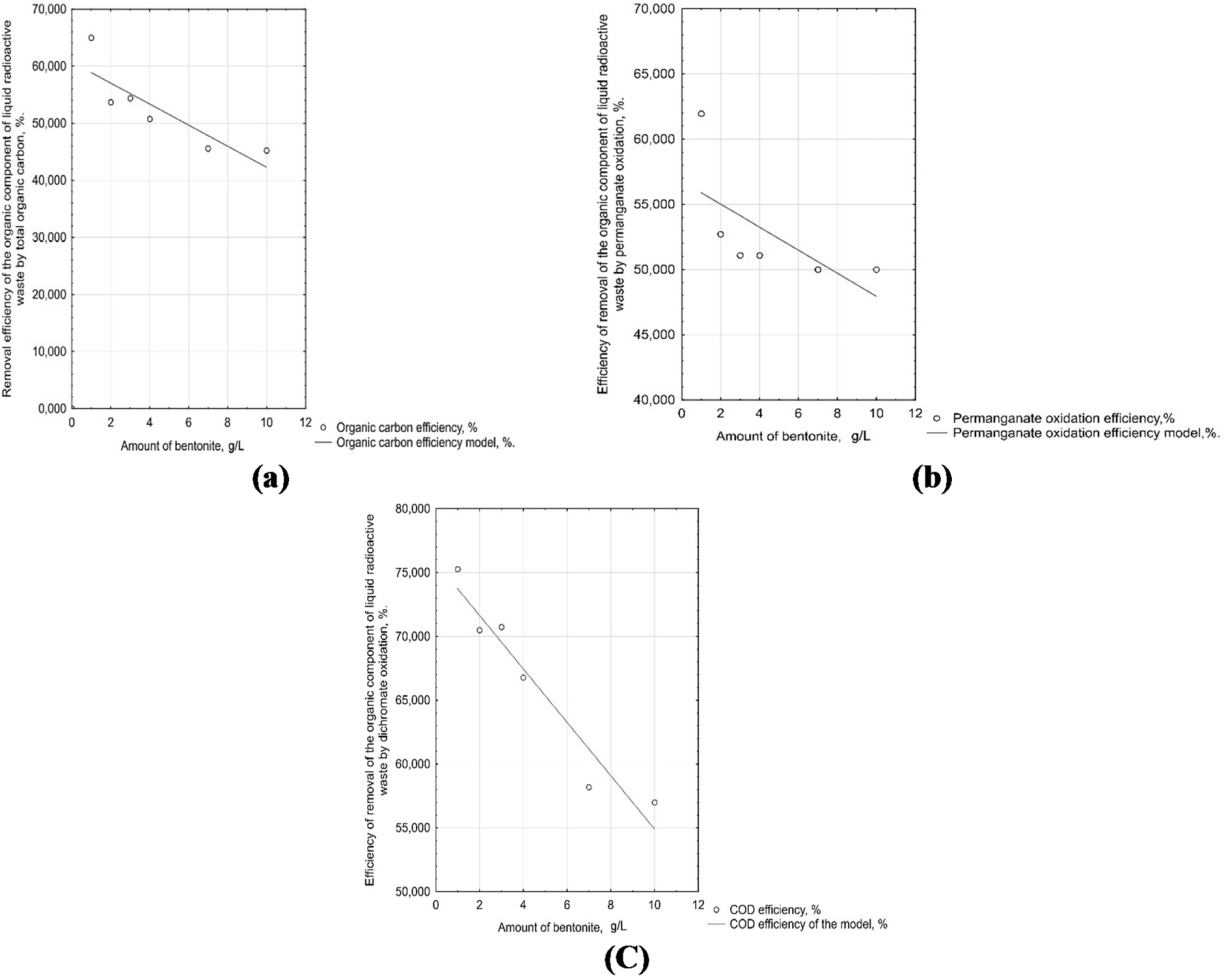
Dependence of the efficiency of removing the organic component of LRW: (**a**) TOC; (**b**) COD(Mn); (**c**) COD(Cr).

Figure 6a shows the efficiency with which organic components are removed from wastewater, depending on the TOC content. The effectiveness of removing organic compounds from wastewater using COD(Mn) (Fig. 6b) and COD(Cr) (Fig. 6c) are demonstrated.

## Results and discussion

A comprehensive correlation analysis was carried out using parametric and non-parametric methods to objectively determine the effects of all reagents on the reduction of the organic component. The results are presented in Tables 6 and 7.

**Table 6:** Parametric pearson correlation.

Reagents	TOC, mgC/L	COD(Mn), mgO_2_/L	COD(Cr), mgO_2_/L
Activated carbon by dry matter, g/L	− 0.95	− 0.98	− 0.97
20% w/v FeCl_3_, ml	− 0.95	− 0.98	− 0.97
Amount of bentonite, g/L	− 0.22	− 0.35	− 0.25

**Table 7:** Non-parametric spearman correlation.

Reagents	TOC, mgC/L	COD(Mn), mgO_2_/L	COD(Cr), mgO_2_/L
Activated carbon by dry matter, g/L	− 0.61	− 0.62	− 0.61
20% w/v FeCl_3_, ml	− 0.61	− 0.62	− 0.61
Amount of bentonite, g/L	0.21	0.22	0.21

During the regression analysis aimed at determining the influence of independent variables affecting the efficiency of organic matter removal, the coefficients of the independent variables in the empirical equations were estimated using the least squares method (LSM). In all obtained empirical models describing the efficiency of organic matter reduction, according to the COD(Mn), COD(Cr), TOC indicators, the regression coefficient of the independent variable FeCl_3_, determined by the LSM, was found to be zero. Therefore, this variable, as well as the influence of FeCl_3_, was formally excluded from further analysis, except for the constant effect of the fixed concentration of the iron-based coagulant, which remained unchanged throughout the experiments.

The models are presented in the form of Eqs. (3–5). These equations use the following symbols: activated carbon (g/L) by dry matter– AV; amount (g/L) of bentonite – KB; efficiency of purification (%) in terms of TOC – Ezov; efficiency of purification (%) according to COD(Mn) – Epo; efficiency of purification (%) according to COD(Cr) – Ehpk;

3 $$\:Ezov\:=\:-1.986\times\:{10}^{-15}+12.144\times\:AV\:-1.841\times\:KB$$

where Ezov is the efficiency of purification by TOC (%); AV is activated carbon by dry substance (g/L); KB is the amount (g/L) of bentonite.

The model characteristics are as follows: multiple correlation *R* = 0.987; multiple determination R^2^ = 0.974; adjusted multiple determination R^2^ = 0.96; Fisher’s F = 73.618; *p* < 0.0007.

4 $$\:Epo=-1.805\times\:{10}^{-14}+11.357\times\:AV-0.884\times\:KB$$

where Epo is the efficiency of purification (%) according to COD(Mn); AV is activated carbon by dry substance (g/L); KB is the amount (g/L) of bentonite.

The model characteristics are as follows: multiple correlation *R* = 0,988; multiple determination R^2^ = 0.976; adjusted multiple determination R^2^ = 0.964; Fisher’s F = 80.516; *p* < 0.0006.

5 $$\:Ehpk=-2.440\times\:{10}^{-14}+15.164\times\:AV-2.091\times\:KB$$

where Еhpk is the efficiency of purification (%) according to COD(Cr); AV is activated carbon by dry substance (g/L); KB is the amount (g/L) of bentonite.

The model characteristics are as follows: multiple correlation *R* = 0.998; multiple determination R^2^ = 0.995; adjusted multiple determination R^2^ = 0.993; Fisher’s F = 427.6996; *p* < 0.000022.

**Fig. 7:**
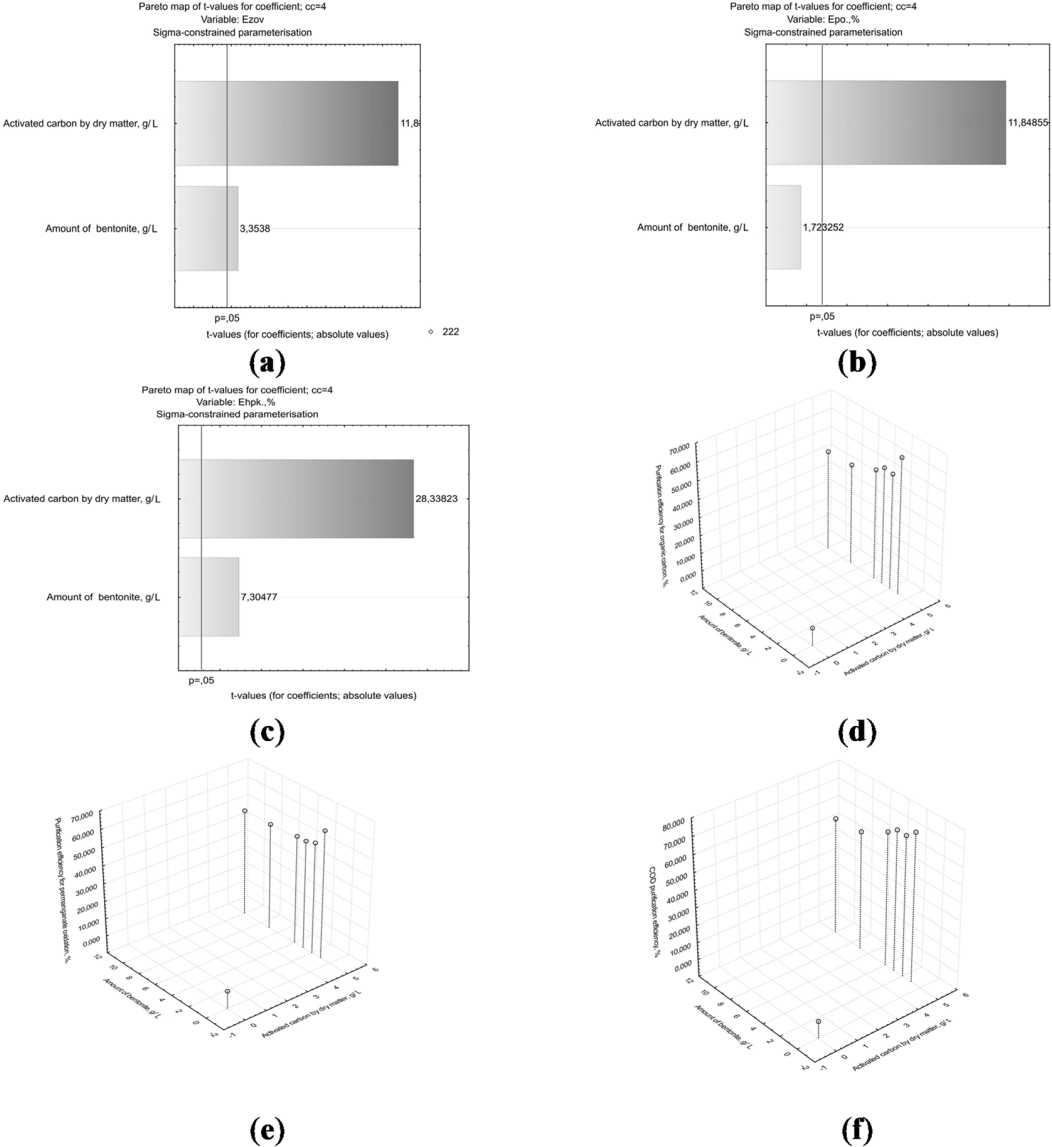
Pareto chart of variable significance in the model of organic component removal efficiency based on: (**a**) TOC, (**b**) COD(Mn); (**c**) COD(Cr). The efficiency of treatment of the organic component of wastewater according to the indicators: (**d**) TOC (%); (**e**) COD(Mn), %; (**f**) COD(Cr), %, activated carbon by dry matter (g/L) and the amount of bentonite (g/L).

The corresponding results are shown in Fig. 7a-f.

We also constructed connection models that reliably predict the conversion of COD(Cr), % into COD(Mn), % and TOC, %.

These are presented in the form of Eqs. (6) and (7).

A model for determining COD(Mn), based on COD(Cr) data:

6 $$\:PO=22.624+0.398\times\:DO-5.999\times\:Ln\left(DO\right)$$

where PO is COD(Mn), mgO_2_/L and DO is COD(Cr), mgO_2_/L.

The model characteristics are as follows: multiple correlation *R* = 0.987; multiple determination R^2^ = 0.975; adjusted multiple determination R^2^ = 0.963; Fisher’s F (2,4) = 78.064; *p* < 0.00062; standard estimation error: 1,1622.

**Fig. 8:**
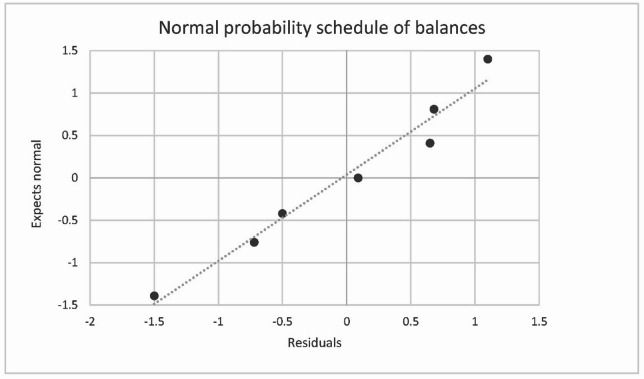
The normality check of total organic carbon residuals by COD(Mn) between real and modelled datasets.

The normality of the residuals distribution is shown in Fig. 8.

A model for determining TOC based on COD(Cr) data:

7 $$\:TOC=0.175\times\:DO+4.029\times\:Ln\left(DO\right)-5.33$$

where is TOC- total organic carbon, mgC/L, DO is COD(Cr), mgO^2^ /L.

The characteristics of the model are as follows: multiple correlation *R* = 0.994; multiple determination R^2^ = 0.988; adjusted multiple determination R^2^ = 0.982; Fisher’s F (2,4) = 166.28; *p* < 0.00014; standard estimation error: 0.75900.

**Fig. 9:**
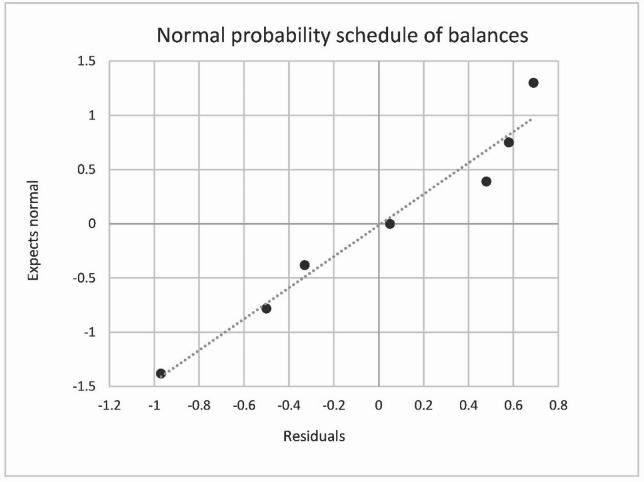
The normality check of organic carbon residuals between real and modelled datasets.

The Fig. 9 shows a graph of the normality of the distribution of TOC residuals.

In this study, we demonstrated the applicability of a combined adsorption–coagulation approach for the removal of organic compounds from liquid radioactive waste simulants. In contrast to the ozonation process, the presented method demonstrated enhanced performance with regard to the removal of organic matter. The values obtained from the analysis indicate a 2.85-fold decrease in total organic carbon (TOC), a 2.63-fold reduction in COD(Mn), and a 4.19-fold decrease in COD(Cr). The achieved purification efficiency of approximately 75% represents a notable improvement.

The findings of the correlation analysis demonstrated that fluctuations in the concentration of bentonite exerted a negligible influence on the efficiency of organic contaminant removal. An increase in the dosage of bentonite in comparison with the optimal level resulted in a decrease in purification efficiency. This phenomenon may be attributed to the stabilisation of colloidal particles. Conversely, the efficiency of FeCl₃ in combination with activated carbon was found to be contingent on the respective concentrations of both reagents. The findings of this study indicate that the efficacy of bentonite is maximised when utilised predominantly as a turbidity agent, thereby facilitating colloid formation, accelerating coagulation, and enhancing sedimentation, as opposed to its utilisation as a primary sorbent.

The calculations, based on the conversion models that have been developed, demonstrate that COD(Cr) values can be recalculated with a high degree of reliability into equivalent COD(Mn) or organic carbon parameters within the experimental range. The proposed models demonstrated satisfactory predictive accuracy within the experimental range, providing a practical tool for interpreting and comparing analytical results in LRW treatment studies.

Given the critical nature of LRW, the issue of its accumulation is particularly pressing in the context of Ukraine’s wartime economy, where the rational use of water resources and low-cost solutions are of particular importance.

The significant volumes of radioactive waste stored in the Chernobyl Nuclear Power Plant exclusion zone pose serious environmental risks amid constant shelling. One promising solution is to convert soluble radionuclides into a solid phase, which significantly reduces waste disposal volume and allows decontaminated water to return to the natural cycle. Despite the existence of advanced purification methods, there is still a lack of systematic research into economically viable technologies for removing organic compounds from radioactive liquid waste, and this study aims to address this gap. Sorption technologies play a key role here by providing a cost-effective way to cement the resulting sludge using geoconcretes (alkaline concretes). These are highly resistant to leaching, environmentally stable and do not require additional heat treatment. Thus, the proposed approach harmoniously combines environmental safety, technological reliability, and economic feasibility in solving the critically important problem of managing LRW.

## Conclusions

The aim of this work is to develop inexpensive, environmentally safe methods of decontaminating radioactive waste that consider water and energy shortages.

This study proved the effectiveness of using bentonite as turbidity agent to promote colloid formation, accelerate coagulation and sedimentation. Based on these findings, the ability of bentonite to reduce the percentage of organic compounds in radioactive waste was confirmed.

Thus, the significant advantage of this combined sorption-coagulation method for removing organic compounds over ozonation has been demonstrated.

The conclusions obtained from this study are as follows:

- The experimental results demonstrated that the combined use of adsorption and coagulation processes reduced the organic content of the wastewater simulant by approximately 3–4 times: TOC decreased by 2.85 times, COD(Mn) by 2.63 times, and chemical oxygen demand COD(Cr) by 4.19 times. Overall, the purification efficiency reached about 75% in terms of dissolved organics.
- The correlation analysis indicated that variations in bentonite concentration had a negligible influence on the reduction of organic contaminants. Moreover, increasing the bentonite dose beyond the optimal level exerted a negative effect on purification efficiency, possibly due to the stabilization of colloidal particles.
- The analysis also revealed that the efficiency of the FeCl_3_ coagulant in combination with activated carbon is significantly affected by their respective concentrations. Therefore, bentonite can be introduced as a turbidity agent to promote colloid formation, accelerate coagulation, and enhance sedimentation.
- Conversion models were developed that allow recalculation of measured COD(Cr) values into equivalent COD(Mn) or organic carbon metrics, with predictive accuracy within the range of the experimental data.

Based on the results of the correlation analysis, it is advisable to consider additional sorbents capable of effectively absorbing surfactants, or introducing different doses and brands of activated carbon, or their combination, in order to improve the results of organic component reduction by this method. Different grades of activated carbon should have different pore sizes to enable the sorption of a wider range of organic molecules.

It is also advisable to test different doses of FeCl_3_ and to check the coagulation effect using aluminium-containing coagulants, such as aluminium oxychloride.

Further development of the work will not only allow us to refine the conclusions obtained, but also to identify new patterns that contribute to a more complete understanding of the problem under study.

## Data Availability

The datasets generated and analysed during the current study are stored at the State Institution “The Institute of Environmental Geochemistry of the National Academy of Sciences of Ukraine” and can be provided up on reasonable request to the corresponding author (dmitriych10@gmail.com). Almost all of the input and output data obtained or analysed during this study are included in this published article.
